# Predictive ability of multi-population genomic prediction methods of phenotypes for reproduction traits in Chinese and Austrian pigs

**DOI:** 10.1186/s12711-024-00915-5

**Published:** 2024-06-26

**Authors:** Xue Wang, Zipeng Zhang, Hehe Du, Christina Pfeiffer, Gábor Mészáros, Xiangdong Ding

**Affiliations:** 1 State Key Laboratory of Animal Biotech Breeding, Key Laboratory of Animal Genetics and Breeding of Ministry of Agriculture and Rural Affairs, National Engineering Laboratory of Animal Breeding, College of Animal Science and Technology, China Agricultural University, Beijing, China; 2 University of Natural Resources and Life Sciences, Vienna, Austria

## Abstract

**Background:**

Multi-population genomic prediction can rapidly expand the size of the reference population and improve genomic prediction ability. Machine learning (ML) algorithms have shown advantages in single-population genomic prediction of phenotypes. However, few studies have explored the effectiveness of ML methods for multi-population genomic prediction.

**Results:**

In this study, 3720 Yorkshire pigs from Austria and four breeding farms in China were used, and single-trait genomic best linear unbiased prediction (ST-GBLUP), multitrait GBLUP (MT-GBLUP), Bayesian Horseshoe (BayesHE), and three ML methods (support vector regression (SVR), kernel ridge regression (KRR) and AdaBoost.R2) were compared to explore the optimal method for joint genomic prediction of phenotypes of Chinese and Austrian pigs through 10 replicates of fivefold cross-validation. In this study, we tested the performance of different methods in two scenarios: (i) including only one Austrian population and one Chinese pig population that were genetically linked based on principal component analysis (PCA) (designated as the “two-population scenario”) and (ii) adding reference populations that are unrelated based on PCA to the above two populations (designated as the “multi-population scenario”). Our results show that, the use of MT-GBLUP in the two-population scenario resulted in an improvement of 7.1% in predictive ability compared to ST-GBLUP, while the use of SVR and KKR yielded improvements in predictive ability of 4.5 and 5.3%, respectively, compared to MT-GBLUP. SVR and KRR also yielded lower mean square errors (MSE) in most population and trait combinations. In the multi-population scenario, improvements in predictive ability of 29.7, 24.4 and 11.1% were obtained compared to ST-GBLUP when using, respectively, SVR, KRR, and AdaBoost.R2. However, compared to MT-GBLUP, the potential of ML methods to improve predictive ability was not demonstrated.

**Conclusions:**

Our study demonstrates that ML algorithms can achieve better prediction performance than multitrait GBLUP models in multi-population genomic prediction of phenotypes when the populations have similar genetic backgrounds; however, when reference populations that are unrelated based on PCA are added, the ML methods did not show a benefit. When the number of populations increased, only MT-GBLUP improved predictive ability in both validation populations, while the other methods showed improvement in only one population.

**Supplementary Information:**

The online version contains supplementary material available at 10.1186/s12711-024-00915-5.

## Background

Today, genomic prediction [1] is widely accepted and has been successfully implemented in animal and plant breeding schemes [2–4]. However, a large reference population size is key to accurate genomic prediction [5, 6]. For small reference populations, e.g., breeds or strains of livestock with small populations, it is rather difficult to obtain a sufficiently large reference population, which limits the predictive ability of genomic prediction. A potential option is to combine multiple populations to construct a large reference population, i.e., multi-population genomic prediction, such as for Holstein populations in the EuroGenomics [7] and North American consortia [6]. The advantages of multi-population genomic prediction have been widely verified and results in dairy cattle suggest that this cost-effective strategy can significantly improve the predictive ability of genomic prediction for numerically small breeds if the reference population is made up of individuals from closely-related breeds [7, 8]. Similarly, studies in pigs have also indicated that, compared with a single reference population, joint reference populations with different genetic backgrounds can further improve the predictive ability of genomic prediction and reduce prediction bias, especially for the prediction of reproductive traits with a low heritability [9]. Likewise, in beef cattle, Bonifazi et al. [10] combined age-adjusted weaning weight phenotypes and genomic data from five Limousin populations using a single-step approach and demonstrated the advantage of using a combined reference population. Cardoso et al. [11] evaluated the potential of improving tick resistance in beef cattle breeds from seven countries through multitrait genomic selection and also demonstrated the benefits of combining data from different breeds.

Currently, the prevailing methods for computing genomic estimated breeding values (GEBV) are the genomic best linear unbiased prediction (GBLUP) method, which is implemented by estimating the variance components and solving the mixed model equations of Henderson [12], and Bayesian methods with different priors using a Markov chain Monte Carlo (MCMC) methods to estimate the required (genetic) parameters [13–15]. Multitrait models are often used for multi-population genomic prediction, which treat the same trait in different populations as different traits and can be used to capture genotype-by-environment (G × E) interactions between populations. However, these models have limitations when there are many environments, as more genetic parameters need to be estimated and model convergence may be difficult to achieve. Thus, it remains difficult to find a method that can perform well when multiple populations with different genetic backgrounds are combined for genomic prediction.

Recently, machine learning (ML) algorithms have been widely and successfully used in gene screening, genotype imputation, genomic prediction, and protein structure and function prediction [16–20]. For genomic prediction, ML algorithms differ from conventional methods in that, as nonparametric methods, they are able to flexibly capture hidden relationships between genotype and phenotype in an adaptive manner, while making few or no specific distributional assumptions for predictors [21]. Accordingly, ML is potentially attractive for handling higher-order nonlinear relationships in high-dimensional genomic data (*e.g.,* epistasis, dominance, or G × E interactions), which are more likely to exist in multi-population genomic prediction. Several studies have shown that ML algorithms, such as support vector machine regression (SVR), kernel ridge regression (KRR), and the AdaBoost ensemble algorithm, have advantages over GBLUP and Bayes B in predicting genomic-enabled prediction values [18, 22, 23].

In spite of growing interest, little research has explored the effectiveness of ML methods for multi-population genomic prediction. Faville et al. [24] compared the prediction performance of GBLUP, kinship using genotyping-by-sequencing (GBS) with depth adjustment (KGD), random forest, and ridge regression models for genomic prediction in five perennial ryegrass populations and found that the predictive ability of KGD and GBLUP were marginally superior or equal to that of ridge regression (RR) and random forest (RF) computational approaches. Moreover, the use of multiple populations without constructing a joint genomic relationship matrix is also challenging for ML methods. Therefore, it is necessary to further explore the feasibility of ML methods for multi-population genomic prediction of phenotypes. In this study, a joint reference population comprised of Chinese and Austrian pig populations was established and the performances of ML methods and of single- and multi-trait GBLUP and Bayesian methods were evaluated in these two populations with similar genetic backgrounds to determine the optimal methods to improve the predictive ability for phenotypes of reproduction traits in Chinese and Austrian pigs.

## Methods

### Ethics statement

The entire procedure for blood sample collection was carried out in strict accordance with the protocol approved by the Animal Care and Use Committee of China Agricultural University (Permit Number: DK996) and Austrian Pig Breeders Association.

### Population and phenotypes

In this study, five Yorkshire pig populations were used. One was provided by the Austria Pig Breeding Association, referred to as 'Austria', and the others were sourced from four breeding farms in China, identified as A, B, C, and D (Table 1). Two reproduction traits, i.e. total number of piglets born (TNB) and number of piglets born alive (NBA), were studied. In the "two-population scenario", we used the Austrian population and Chinese pig population A, which were shown to be genetically linked based on principal component analysis (PCA) of genotypes, for joint genomic prediction. In the “multi-population scenario”, we added data from the other three Chinese populations, which had more dispersed genetic backgrounds, to the Austrian and A populations to assess the impact of expanding the reference population size on the predictive ability for the Austrian and the A population. This scenario was termed the “multi-population scenario”. Because the main aim of this study was to explore whether adding unrelated populations could improve the prediction ability for two genetically connected populations, we did not make predictions for the other populations in the multi-population scenario.

**Table 1:** Summary of the five Yorkshire populations, number of genotyped individuals and heritability estimates (h^2^)

SNP panels	Population	Trait^a^	σ^2^_*a*_	σ^2^_*e*_	h^2^(SE)	Number of records	Birth year	Genotyped animals
Austria 50K	Austria	TNB	0.71	6.69	0.09 (0.03)	3713	2006–2017	591
		NBA	0.49	5.71	0.08 (0.03)			
PorcineSNP50 BeadChip; GenoBaits Porcine SNP 50 K	A	TNB	1.26	8.95	0.12 (0.03)	2841	2016–2020	742
		NBA	0.99	7.49	0.11 (0.03)			
PorcineSNP50 BeadChip	C	TNB	1.62	11.46	0.12 (0.02)	4144	2015–2018	1153
		NBA	1.55	11.45	0.12 (0.02)			
	B	TNB	0.59	7.77	0.07 (0.01)	2209	2015–2018	550
		NBA	0.37	6.73	0.05 (0.01)			
	D	TNB	1.05	11.53	0.08 (0.03)	1209	2018–2019	684
		NBA	0.58	10.96	0.05 (0.02)			

^a^*TNB* total number of piglets born, *NBA* number of piglets born alive, *SE* standard error

### Derivation of corrected phenotypes

To avoid double-counting parental information, corrected phenotypic values (${\mathbf{y}}_{\mathbf{c}}$) derived from pedigree-based estimated breeding values (EBV) were used as response variables in all genomic prediction analyses [25]. For this purpose, single-trait repeatability models were used to estimate EBV for TNB and NBA and genetic parameters, separately for each population. In the model, the fixed effect was herd-year-season, and the random effects were additive genetics ($\mathbf{a}$), permanent environment (${\mathbf{p}}_{\mathbf{e}}$), and residuals ($\mathbf{e}$). The random effects were assumed to have the following distributions: $\mathbf{a}\sim N(\mathbf{0},\mathbf{A}{\upsigma }_{a}^{2})$, ${\mathbf{p}}_{\mathbf{e}}\sim N(\mathbf{0},\mathbf{I}{\upsigma }_{pe}^{2})$, and $\mathbf{e}\sim N(\mathbf{0},\mathbf{I}{\upsigma }_{e}^{2})$, where $\mathbf{A}$ is the pedigree-based relationship matrix, $\mathbf{I}$ is the identity matrix, and ${\upsigma }_{a}^{2}$, ${\upsigma }_{pe}^{2}$, and ${\upsigma }_{e}^{2}$ are the variances of additive genetic effects, permanent environment effects of sows, and residuals, respectively. The $\mathbf{A}$ matrix for each population was constructed separately using the pedigree of each population, and a total of 14,118 animals were traced across the five populations. The number of generations and full-sib and half-sib families for each population are listed in Additional file 1: Table S1. The estimated genetic parameters are in Table 1. EBV were calculated using the DMUAI procedure in the DMU software [26]. The ${\mathbf{y}}_{\mathbf{c}}$ values were calculated as the EBV plus the average estimated residuals for multiple parities of a sow following Song et al. [27].

### Genotype data and imputation

**Fig. 1:**
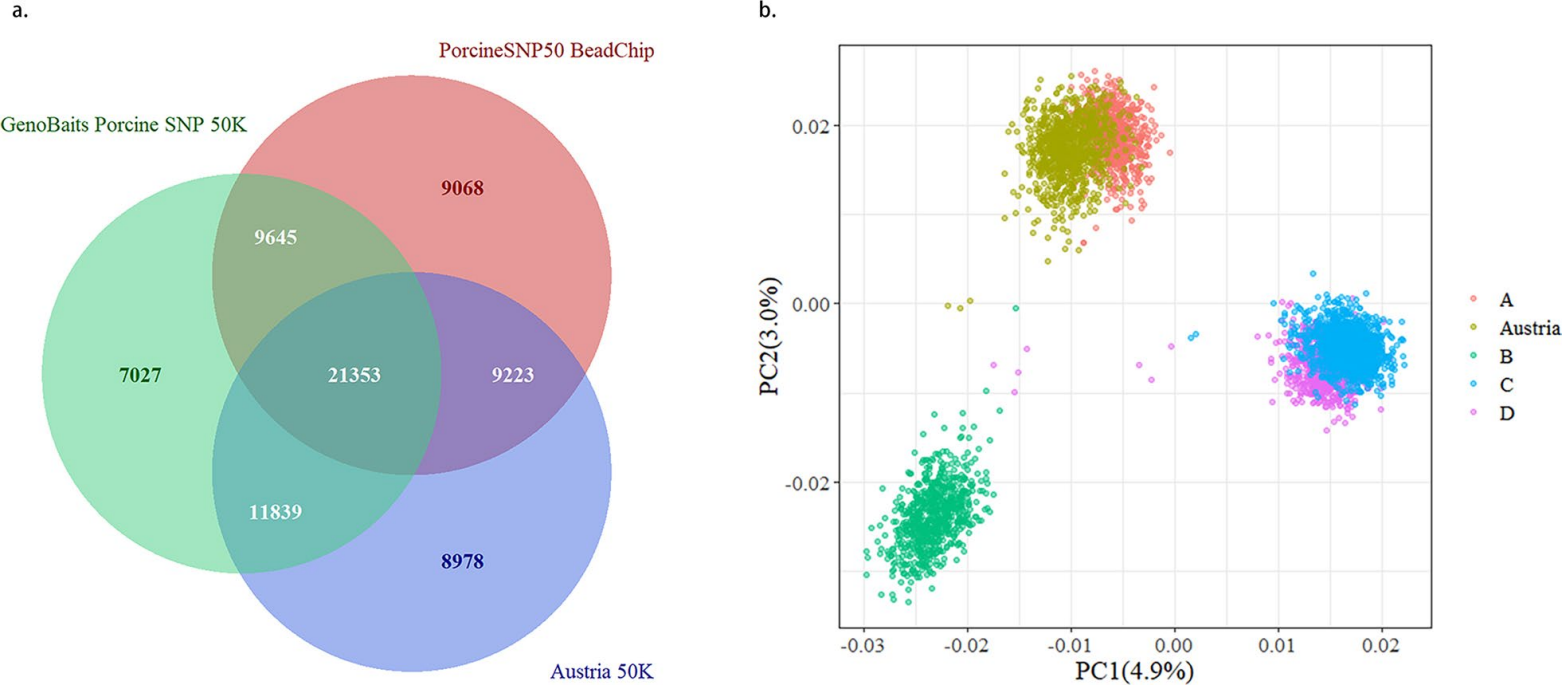
Venn diagram of the number of shared SNPs between panels (**a**) and principal component analysis of the Austrian and Chinese Yorkshire populations (**b**). *PC1* first principal component, *PC2* second principal component

In this study, the Austrian pigs were genotyped using a customized 50 K SNP panel (59,319 SNPs, Austria 50 K), while all Chinese populations were genotyped using the PorcineSNP50 BeadChip (Illumina, CA, USA) (50,697 SNPs, PorcineSNP50) and population A was also genotyped using the GenoBaits Porcine SNP 50 K panel (Molbreeding, China) (52,000 SNPs, GBTS50K). As shown in Fig. 1a, 31,062 SNPs were shared between the Austria 50 K and PorcineSNP50 panels, 33,192 SNPs between the Austria 50 K and GBTS50K panels, and 30,998 SNPs between the PorcineSNP50 and GBTS50K panels. In total, 3720 individuals were genotyped, and the number of genotyped individuals in each population is in Table 1. The individuals genotyped with the Austria 50 K and PorcineSNP50 panels were imputed to GBTS50K using the Beagle 5.0 software [28], with the reference population for imputation comprising 4839 Yorkshire pigs from multiple farms in China (including the four farms in this study). The theoretical imputation accuracy was assessed by the dosage R-squared (DR^2^), which is the estimated squared correlation between the estimated allele dose and the true allele dose that is calculated in Beagle 5.0 [28].

No common individuals were genotyped by both the Austria 50 K and the other two SNP panels. Thus, to further evaluate the practical effect of genotype imputation, first, all the non-missing SNPs (1112 SNPs) among the SNPs shared between the Austria 50 K and GBTS50K panels were set as missing. In addition, 2000 SNPs were randomly selected from the 29,488 shared non-missing SNPs of the PorcineSNP50 and GBTS50K panels and set as missing; then, Beagle 5.0 was used for re-imputation and the genotype concordance rate (CR) was calculated to evaluate imputation accuracy. After imputation, the PLINK software [29] was used to remove SNPs with a minor allele frequency (MAF) lower than 0.05 and a call rate less than 0.90 and animals with a call rate less than 0.90. After genotype quality control, all remaining individuals (3720) and 47,734 SNPs on autosomes were retained for further analysis.

### Principal component analysis and linkage disequilibrium

To analyse the population structure of the five populations, PCA was performed on the SNP genotypes using the GCTA software [30] and a matrix of eigenvectors, in descending order, representing the principal components (PC), with PC1 having the largest eigenvalue, was created. The LD between each pair of SNPs was measured as ${\text{r}}_{\text{LD}}$ and ${\text{r}}_{\text{LD}}^{2}$ (i.e. the square of ${\text{r}}_{\text{LD}}$) [31], using

$${\text{r}}_{{{\text{LD}}}} = \frac{{{\text{f}}\left( {{\text{AB}}} \right) - {\text{f}}({\text{A}}){\text{f}}({\text{B}})}}{{\sqrt {{\text{f}}({\text{A}}){\text{f}}({\text{a}}){\text{f}}({\text{B}}){\text{f}}({\text{b}})} }},$$

 where $\text{f}\left(\text{AB}\right)$, $\text{f}(\text{A})$, $\text{f}(\text{B})$, $\text{f}(\text{a})$, and $\text{f}(\text{b})$ are the observed frequencies of haplotype AB and alleles A, B, a, and b, respectively. The average ${\text{r}}_{\text{LD}}^{2}$ across all chromosomes was calculated for each population, and the consistency of LD between the Austria and A populations and with other populations was measured using the correlation of ${\text{r}}_{\text{LD}}$ values of pairs of adjacent SNPs on each autosome.

### Statistical models

Single-trait GBLUP (ST-GBLUP), multitrait GBLUP (MT-GBLUP), BayesHE, and three ML methods (SVR, KRR and Adaboost.R2) were evaluated. For all methods, the response variables were the corrected phenotypes ${\mathbf{y}}_{\mathbf{c}}$ and the independent variables for the three ML methods were the vectors of SNP genotypes, encoded as 0, 1, and 2.

#### Single-trait GBLUP (ST-GBLUP)

The model used for ST-GBLUP was:

$${\mathbf{y}}_{\mathbf{c}}=1\upmu +\mathbf{Z}\mathbf{a}+\mathbf{e},$$

where ${\mathbf{y}}_{\mathbf{c}}$ is the vector of corrected phenotypes of genotyped individuals; $\upmu$ is the overall mean, and $\mathbf{1}$ is a vector of ones; $\mathbf{a}$ is the vector of additive genetic effects, assumed distributed $N(\mathbf{0},\mathbf{G}{\sigma }_{a}^{2})$, where ${\sigma }_{a}^{2}$ is the additive genetic variance and $\mathbf{G}$ is the genomic relationship matrix; $\mathbf{Z}$ is the incidence matrix allocating records to $\mathbf{a}$; $\mathbf{e}$ is the vector of random errors, assumed distributed $N(\mathbf{0},\mathbf{I}{\sigma }_{e}^{2})$, where $\mathbf{I}$ is the identity matrix and ${\sigma }_{e}^{2}$ is the residual variance. The $\mathbf{G}$ matrix was constructed following the first method proposed by VanRaden [32]. The ST-GBLUP model was fitted using the DMU software [26] and the variance components were estimated using the average information restricted maximum likelihood (AI-REML) algorithm implemented of the DMUAI procedure.

#### Multitrait GBLUP (MT-GBLUP)

For the two-population scenario, the MT-GBLUP model was:

$$\left[\begin{array}{c}{\mathbf{y}}_{\mathbf{c}1}\\ {\mathbf{y}}_{\mathbf{c}2}\end{array}\right]=\left[\begin{array}{cc}{\mathbf{1}}& {\mathbf{0}}\\ {\mathbf{0}}& {\mathbf{1}}\end{array}\right]\left[\begin{array}{c}{\upmu }_{1}\\ {\upmu }_{2}\end{array}\right]+\left[\begin{array}{cc}{\mathbf{Z}}_{1}& {\mathbf{0}}\\ {\mathbf{0}}& {\mathbf{Z}}_{2}\end{array}\right]\left[\begin{array}{c}{\mathbf{a}}_{1}\\ {\mathbf{a}}_{2}\end{array}\right]+\left[\begin{array}{c}{\mathbf{e}}_{1}\\ {\mathbf{e}}_{2}\end{array}\right]\boldsymbol{ },$$

where $$\left[\begin{array}{c}{\mathbf{y}}_{\mathbf{c}1}\\ {\mathbf{y}}_{\mathbf{c}2}\end{array}\right]$$ are the vectors of corrected phenotypes for trait 1 and trait 2 (the same trait in the A and Austrian populations); $$\left[\begin{array}{c}{\upmu }_{1}\\ {\upmu }_{2}\end{array}\right]$$ is the vector of overall means for trait 1 and trait 2; $$\left[\begin{array}{c}{\mathbf{a}}_{1}\\ {\mathbf{a}}_{2}\end{array}\right]$$ are the vectors of additive genetic effects of the two traits, assumed distributed $N\left(\mathbf{0}, \mathbf{M}{\mathbf{\otimes}} \mathbf{G}\right)$, where $$\mathbf{M}=\left[\begin{array}{cc}{\sigma }_{a1}^{2}& {\sigma }_{a12}\\ {\sigma }_{a12}& {\sigma }_{a2}^{2}\end{array}\right]$$ represents the genetic variance and covariance matrix of the two traits; ${\mathbf{Z}}_{1}$ and ${\mathbf{Z}}_{2}$ are the incidence matrices allocating records to ${\mathbf{a}}_{1}$ and${\mathbf{a}}_{2}$, respectively $$;\left[\begin{array}{c}{\mathbf{e}}_{1}\\ {\mathbf{e}}_{2}\end{array}\right]$$ are the vectors of random residual errors, assumed to be normally distributed $N(0,\mathbf{R}{\mathbf{\otimes}} \mathbf{I})$, where $\mathbf{I}$ is the identity matrix and $$\mathbf{R}=\left[\begin{array}{cc}{\sigma }_{e1}^{2}& {\sigma }_{e12}\\ {\sigma }_{e12}& {\sigma }_{e2}^{2}\end{array}\right]$$ is the residual variance and covariance matrix.

For the multi-population scenario, the MT-GBLUP model can be expressed as:

$$\left[\begin{array}{c}{\mathbf{y}}_{\mathbf{c}1}\\ {\mathbf{y}}_{\mathbf{c}2}\\ {\mathbf{y}}_{\mathbf{c}3}\\ {\mathbf{y}}_{\mathbf{c}4}\\ {\mathbf{y}}_{\mathbf{c}5}\end{array}\right]=\left[\begin{array}{ccccc}{\mathbf{1}}& {\mathbf{0}}& {\mathbf{0}}& {\mathbf{0}}& {\mathbf{0}}\\ {\mathbf{0}}& {\mathbf{1}}& {\mathbf{0}}& {\mathbf{0}}& {\mathbf{0}}\\ {\mathbf{0}}& {\mathbf{0}}& {\mathbf{1}}& {\mathbf{0}}& {\mathbf{0}}\\ {\mathbf{0}}& {\mathbf{0}}& {\mathbf{0}}& {\mathbf{1}}& {\mathbf{0}}\\ {\mathbf{0}}& {\mathbf{0}}& {\mathbf{0}}& {\mathbf{0}}& {\mathbf{1}}\end{array}\right]\left[\begin{array}{c}{{\varvec{\upmu}}}_{1}\\ {{\varvec{\upmu}}}_{2}\\ {{\varvec{\upmu}}}_{3}\\ {{\varvec{\upmu}}}_{4}\\ {{\varvec{\upmu}}}_{5}\end{array}\right]+\left[\begin{array}{ccccc}{\mathbf{Z}}_{1}& {\mathbf{0}}& {\mathbf{0}}& {\mathbf{0}}& {\mathbf{0}}\\ {\mathbf{0}}& {\mathbf{Z}}_{2}& {\mathbf{0}}& {\mathbf{0}}& {\mathbf{0}}\\ {\mathbf{0}}& {\mathbf{0}}& {\mathbf{Z}}_{3}& {\mathbf{0}}& {\mathbf{0}}\\ {\mathbf{0}}& {\mathbf{0}}& {\mathbf{0}}& {\mathbf{Z}}_{4}& {\mathbf{0}}\\ {\mathbf{0}}& {\mathbf{0}}& {\mathbf{0}}& {\mathbf{0}}& {\mathbf{Z}}_{5}\end{array}\right]\left[\begin{array}{c}{\mathbf{a}}_{1}\\ {\mathbf{a}}_{2}\\ {\mathbf{a}}_{3}\\ {\mathbf{a}}_{4}\\ {\mathbf{a}}_{5}\end{array}\right]+\left[\begin{array}{c}{\mathbf{e}}_{1}\\ {\mathbf{e}}_{2}\\ {\mathbf{e}}_{3}\\ {\mathbf{e}}_{4}\\ {\mathbf{e}}_{5}\end{array}\right] ,$$

where ${\left[{\mathbf{y}}_{\mathbf{c}1},\boldsymbol{ }{\mathbf{y}}_{\mathbf{c}2},\boldsymbol{ }{\mathbf{y}}_{\mathbf{c}3},\boldsymbol{ }{\mathbf{y}}_{\mathbf{c}4},\boldsymbol{ }{\mathbf{y}}_{\mathbf{c}5}\right]}^{\text{T}}$ are the vectors of corrected phenotypes for traits 1 to 5 (the same trait in the five populations); ${\left[{{\varvec{\upmu}}}_{1},\boldsymbol{ }{{\varvec{\upmu}}}_{2},\boldsymbol{ }{{\varvec{\upmu}}}_{3},\boldsymbol{ }{{\varvec{\upmu}}}_{4},\boldsymbol{ }{{\varvec{\upmu}}}_{5}\right]}^{\text{T}}$ are the vectors of overall means for traits 1 to 5; ${\left[{\mathbf{a}}_{1},\boldsymbol{ }{\mathbf{a}}_{2},\boldsymbol{ }{\mathbf{a}}_{3},\boldsymbol{ }{\mathbf{a}}_{4},\boldsymbol{ }{\mathbf{a}}_{5}\right]}^{\text{T}}$ are the vectors of additive genetic effects of the five traits, assumed distributed $N\left(\mathbf{0}, \mathbf{M}{\mathbf{\otimes}} \mathbf{G}\right)$, where

$$\mathbf{M}=\left[\begin{array}{ccccc}{\sigma }_{a1}^{2}& {\sigma }_{a12}& {\sigma }_{a13}& {\sigma }_{a14}& {\sigma }_{a15}\\ {\sigma }_{a12}& {\sigma }_{a2}^{2}& {\sigma }_{a23}& {\sigma }_{a24}& {\sigma }_{a25}\\ {\sigma }_{a13}& {\sigma }_{a23}& {\sigma }_{a3}^{2}& {\sigma }_{a34}& {\sigma }_{a35}\\ {\sigma }_{a14}& {\sigma }_{a24}& {\sigma }_{a34}& {\sigma }_{a4}^{2}& {\sigma }_{a45}\\ {\sigma }_{a15}& {\sigma }_{a25}& {\sigma }_{a35}& {\sigma }_{a45}& {\sigma }_{a5}^{2}\end{array}\right]$$

is the genetic variance and covariance matrix of the five traits; ${\mathbf{Z}}_{1}$ to ${\mathbf{Z}}_{5}$ are the incidence matrices allocating records to ${\mathbf{a}}_{1}$ to ${\mathbf{a}}_{5}$, respectively$;$ ${\left[{\mathbf{e}}_{1},\boldsymbol{ }{\mathbf{e}}_{2},\boldsymbol{ }{\mathbf{e}}_{3},\boldsymbol{ }{\mathbf{e}}_{4},\boldsymbol{ }{\mathbf{e}}_{5}\right]}^{\text{T}}$ are the vectors of random residual errors, assumed distributed $N\left(\mathbf{0},\mathbf{R}{\mathbf{\otimes}} \mathbf{I}\right)$, where

$$\mathbf{R}=\left[\begin{array}{ccccc}{\sigma }_{e1}^{2}& {\sigma }_{e12}& {\sigma }_{e13}& {\sigma }_{e14}& {\sigma }_{e15}\\ {\sigma }_{e12}& {\sigma }_{e2}^{2}& {\sigma }_{e23}& {\sigma }_{e24}& {\sigma }_{e25}\\ {\sigma }_{e13}& {\sigma }_{e23}& {\sigma }_{e3}^{2}& {\sigma }_{e34}& {\sigma }_{e35}\\ {\sigma }_{e14}& {\sigma }_{e24}& {\sigma }_{e34}& {\sigma }_{e4}^{2}& {\sigma }_{e45}\\ {\sigma }_{e15}& {\sigma }_{e25}& {\sigma }_{e35}& {\sigma }_{e45}& {\sigma }_{e5}^{2}\end{array}\right]$$

represents the residual variance and covariance matrix.

For the MT-GBLUP methods, the $\mathbf{G}$ matrix was also constructed according to the first method proposed by VanRaden [32], and the variance components were calculated using the DMU software [26]. For the multi-population scenario, (co)variance component parameters were estimated using bivariate analyses for each pair of populations with the AI-REML algorithm of the DMU software [26], and the (co)variance matrix was converted into a positive definite matrix using a bending procedure [33]. The R package mbend was used to implement unweighted bending of variance components [34], with the other parameters set to default values (i.e. max.iter, small.positive and method were 10000, 0.0001 and “hj”, respectively). Given that the variance components in both scenarios were estimated using the complete dataset, we calculated the genetic correlation for each trait among the five populations based on the variance component results of MT-GBLUP in the multi-population scenario. The estimated values of genetic (co)variance and genetic correlation between pairs of the five populations are in Additional file 1: Table S2. Genetic correlations were calculated as $\frac{{\sigma }_{a12}}{\sqrt{{\sigma }_{a1}^{2}{\sigma }_{a2}^{2}}}$.

#### Bayes Horseshoe

Bayes horseshoe (Bayes HE), developed by Shi et al. [35], is a Bayesian model based on global–local priors (i.e., the global parameter shrinks the marker effects to zero, whereas the local parameter allows a marker to escape from shrinkage) to increase the flexibility and adaptability of hyperparameter estimation. BayesHE exhibited the highest or second highest predictive ability compared with traditional Bayesian methods such as BayesA and BayesB [35]. The model fitted for Bayes HE was:

$$\begin{array}{c}{\mathbf{y}}_{\mathbf{c}}={\mathbf{1}}\upmu +\sum_{k=1}^{m}{\mathbf{x}}_{k}{\upbeta }_{k}+\mathbf{e},\end{array}$$

where ${\mathbf{y}}_{\mathbf{c}}$ is the vector of corrected phenotypes of genotyped animals, $\upmu$ is the overall mean, ${\mathbf{x}}_{k}$ is the genotype vector of the *k*-th marker, and *m* is the number of markers; ${\upbeta }_{k}$ is the allele substitution effect of the *k*-th marker, assumed to be distributed as ${\upbeta }_{k}\sim N(0,{\uplambda }_{k}^{2}{\uptau }^{2})$, where the local parameter ${\uplambda }_{k}$ follows a half-t distribution of ${\uplambda }_{k}\sim {\text{half}-\text{t}}^{+}(\upupsilon ,1)$ ($\upupsilon \sim \text{G}\left(\text{a},\text{c}\right)$) and the global parameter $\uptau$ follows a positive half-Cauchy distribution of $\uptau \sim {\text{C}}^{+}(0,{\text{N}}^{-1})$; $\mathbf{e}$ is the vector of random residuals, assumed distributed as $N(\mathbf{0},\mathbf{I}{\upsigma }_{e}^{2})$. In this study, the first form of BayesHE (BayesHE1) was used, in which a is equal to 4 and c is equal to 1 [35]. Using in-house scripts written in Fortran 95 [35], the Markov chain Monte Carlo (MCMC) chain was run for 50,000 cycles, with the first 20,000 cycles discarded as burn-in, and every 50th sample of the remaining 30,000 iterations was saved for inferring posterior statistics.

#### Support vector regression

SVR is a classic algorithm for dealing with regression problems in machine learning. It can use the nonlinear kernel function (e.g. radial basis function (RBF) kernel) to map the input data of the original space into the high-dimensional kernel space and model and predict in the high-dimensional kernel space [36]. Therefore, we can construct linear models in the feature space to address regression problems. SVR was fitted using the following model:

$$\text{f}\left(\mathbf{x}\right)=\text{b}+{\mathbf{h}\left(\mathbf{x}\right)}^{\text{T}}\mathbf{w},$$

where $\text{f}(\mathbf{x})$ is the predicted value, ${\mathbf{h}(\mathbf{x})}^{\text{T}}$ denotes the kernel function, representing a nonlinear transformation of the predictor variables in $\mathbf{x}$ (i.e., genotype vector), $\mathbf{w}$ is the vector of weights, and $\text{b}$ is the intercept. In the context of 'ε-insensitive' SVM regression, the loss was calculated only when the absolute value of the discrepancy between $\text{f}({\mathbf{x}}_{\text{i}})$ and ${\text{y}}_{\text{i}}$ exceeded some constant ($\upvarepsilon$). The SVR problem can be formalized as [36]:

$$\underset{\text{w},\text{b}}{\textrm{min}}\frac{1}{2}{\Vert \mathbf{w}\Vert }^{2}+\text{C}\sum\nolimits_{\text{i}=1}^{\textrm{n}}{\text{V}}_{\upvarepsilon }\left({\text{y}}_{\textrm{ci}}-\text{f}\left({\mathbf{x}}_{\text{i}}\right)\right),$$

where $${\text{V}}_{\upvarepsilon }\left(\text{z}\right)=\left\{\begin{array}{c}0, \quad if \left|\text{z}\right|<\varepsilon ;\\ \left|\text{z}\right|-\varepsilon , otherwise.\end{array} ,\right.$$

with $\text{C}$ being the regularization constant, ${\mathbf{y}}_{\mathbf{c}}$ the vector of corrected phenotypes, ||·|| is the norm in Hilbert space, $\text{n}$ is the sample size, $\text{z}$ is the error (i.e., ${\text{y}}_{\text{ci}}-\text{f}\left({\mathbf{x}}_{\text{i}}\right)$), and ${\text{V}}_{\upvarepsilon }$ is the ‘*ε*-insensitive’ loss. After optimization, SVR can be expressed as:

$$\text{f}\left(\mathbf{x}\right)=\sum\nolimits_{\text{i}=1}^{\text{n}}\left({\widehat{\text{a}}}_{\text{i}}-{\text{a}}_{\text{i}}\right)\text{K}\left(\mathbf{x},{\mathbf{x}}_{\text{i}}\right)+\text{b} ,$$

where ${\widehat{\text{a}}}_{\text{i}}$ and ${\text{a}}_{\text{i}}$ denote positive weights assigned to each observation and estimated from the data, and $\text{K}\left(\mathbf{x},{\mathbf{x}}_{\text{i}}\right)$ represents the inner product of $\mathbf{x}$ (a new input data point) and ${\mathbf{x}}_{\text{i}}$ (the i-th data point in the training dataset) after being mapped to a high-dimensional space through a kernel function (i.e. $\text{K}\left(\mathbf{x},{\mathbf{x}}_{\text{i}}\right)=\mathbf{h}(\mathbf{x})\cdot {\mathbf{h}({\mathbf{x}}_{\text{i}})}^{\text{T}}$). A grid search was used to identify the optimal kernel function and the hyperparameters for $\text{C}$ and gamma (the parameter controlling kernel width in the RBF kernel).

#### Kernel ridge regression

Kernel ridge regression (KRR), as a nonlinear regression method, can be used to effectively mine the nonlinear structure of data [37]. Like SVR, KRR uses a nonlinear kernel function (${\varvec{\upphi}}({\mathbf{x}}_{\text{i}})$) to map the original data into a high-dimensional feature space and then builds a ridge regression model in the feature space for prediction. The linear regression model is expressed as ${\text{y}}_{\text{i}}={{\varvec{\upbeta}}}^{\text{T}}{\varvec{\upphi}}({\mathbf{x}}_{\text{i}})$, where ${\varvec{\upbeta}}$ denotes the weight vector. KRR employs regularized least squares to ascertain the weight vector ${\varvec{\upbeta}}$ by minimizing the following objective function [37]:

$${\text{minL}}_{\text{KRR}}=\frac{1}{2}{\Vert {\varvec{\upbeta}}\Vert }^{2}+\frac{1}{2\uplambda }\sum\nolimits_{\text{i}=1}^{\text{n}}{({\text{y}}_{\text{ci}}-{{\varvec{\upbeta}}}^{\text{T}}{\varvec{\upphi}}({\mathbf{x}}_{\text{i}}))}^{2} ,$$

where $\uplambda$ is the regularization constant. By computing the derivative of ${\text{L}}_{\text{KRR}}$ with respect to ${\varvec{\upbeta}}$ and setting the resulting equations to zero, the resultant weight vector ${\varvec{\upbeta}}$ is determined as follows:

$${\varvec{\upbeta}}={({{\varvec{\upphi}}}^{\text{T}}{\varvec{\upphi}}+\uplambda \mathbf{I})}^{-1}{{\varvec{\upphi}}}^{\text{T}}{\mathbf{y}}_{\mathbf{c}}\boldsymbol{ },$$

where ${\varvec{\upphi}}$ contains the mapped samples ${\varvec{\upphi}}({\mathbf{x}}_{\text{i}})$ in its rows. In other words, ${\varvec{\upphi}}({\mathbf{x}}_{\text{i}})$ represents the vector obtained by applying the feature mapping to a single data point ${\mathbf{x}}_{\text{i}}$, while ${\varvec{\upphi}}$ represents the feature matrix of the entire dataset. $\mathbf{I}$ is the identity matrix. Using the representer’s theorem, ${\varvec{\upbeta}}$ can be expressed in relation to the dual weights ${\upalpha }$ as:

$${\varvec{\upbeta}}= \sum\nolimits_{{i\, = \,1}}^{n}{\upalpha }_{\text{i}}{\varvec{\upphi}}\left({\mathbf{x}}_{\text{i}}\right)={{\varvec{\upphi}}}^{\text{T}}{\upalpha }.$$

Hence, the closed-form solution for the dual weight ${\upalpha }$ is obtained as follows:

$${\upalpha }={({{\varvec{\upphi}}}^{\text{T}}{\varvec{\upphi}}+\uplambda \mathbf{I})}^{-1}{\mathbf{y}}_{\mathbf{c}}={(\mathbf{K}+\uplambda \mathbf{I})}^{-1}{\mathbf{y}}_{\mathbf{c}}\boldsymbol{ },$$

where $\mathbf{K}$ is the kernel matrix (i.e., Gram matrix), and ${\text{K}}_{\text{ij}}=\text{K}\left({\mathbf{x}}_{\text{i}},{\mathbf{x}}_{\text{j}}\right)={\varvec{\upphi}}({\mathbf{x}}_{\text{i}})\cdot {{\varvec{\upphi}}({\mathbf{x}}_{\text{j}})}^{\text{T}}$. If the number of training samples is n, the kernel matrix can be expressed as:

$$\mathbf{K}={\left[\begin{array}{ccc}\text{K}({\mathbf{x}}_{1},{\mathbf{x}}_{1})& \text{K}({\mathbf{x}}_{1},{\mathbf{x}}_{2})& \begin{array}{cc}\cdots & \text{K}({\mathbf{x}}_{1},{\mathbf{x}}_{\text{n}})\end{array}\\ \text{K}({\mathbf{x}}_{2},{\mathbf{x}}_{1})& \text{K}({\mathbf{x}}_{2},{\mathbf{x}}_{2})& \begin{array}{cc}\cdots & \text{K}({\mathbf{x}}_{2},{\mathbf{x}}_{\text{n}})\end{array}\\ \begin{array}{c}\vdots \\ \text{K}({\mathbf{x}}_{\text{n}},{\mathbf{x}}_{1})\end{array}& \begin{array}{c}\vdots \\ \text{K}({\mathbf{x}}_{\text{n}},{\mathbf{x}}_{2})\end{array}& \begin{array}{cc}\begin{array}{c}\vdots \\ \cdots \end{array}& \begin{array}{c}\vdots \\ \text{K}({\mathbf{x}}_{\text{n}},{\mathbf{x}}_{\text{n}})\end{array}\end{array}\end{array}\right]}_{\text{n}\times \text{n}}$$

Ultimately, given a new test sample ${\mathbf{x}}_{\text{i}}$ (i.e. the genotype vector of individual i), the predicted output is derived using dual weights, and the similarity between the test sample ${\mathbf{x}}_{\text{i}}$ and all training samples is employed for prediction. Thus, the expression of KRR is:

$$\text{y}\left({\mathbf{x}}_{\text{i}}\right)={\mathbf{k}}{\mathbf{^{\prime}}{\left(\mathbf{K}+\uplambda \mathbf{I}\right)}^{-1}{\mathbf{y}}_{\mathbf{c}}}\boldsymbol{ },$$

where $\text{y}\left({\mathbf{x}}_{\text{i}}\right)$ is the predicted value of sample ${\mathbf{x}}_{\text{i}}$, ${\mathbf{k}}^{\mathbf{^{\prime}}}=\mathbf{K}({\mathbf{x}}_{\text{i}},{\mathbf{x}}_{\text{j}})$ ($\text{j}$ = 1,2,3,…,n), and the expanded form of ${\mathbf{k}}^{\mathbf{^{\prime}}}$ is:

$${\mathbf{k}}^{\mathbf{^{\prime}}}={\left[\begin{array}{c}\begin{array}{c}\text{K}({\mathbf{x}}_{\text{i}},{\mathbf{x}}_{1})\\ \text{K}({\mathbf{x}}_{\text{i}},{\mathbf{x}}_{2})\end{array}\\ \vdots \\ \text{K}({\mathbf{x}}_{\text{i}},{\mathbf{x}}_{\text{n}})\end{array}\right]}^{\text{T}}$$

Like SVR, a grid search was used to find the optimal kernel function, $\uplambda ,$ and the RBF kernel parameter gamma.

#### AdaBoost.R2

The expression for AdaBoost.R2 can be written as [38]:

$${\text{f}}\left( {\mathbf{x}} \right) = {{\mathop \sum \nolimits_{{t = 1}}^{M} \left( {{\text{log}}\frac{1}{{{\upvarepsilon }_{{t}} }}} \right){\text{f}}_{{t}} \left( {\mathbf{x}} \right)} \mathord{\left/ {\vphantom {{\mathop \sum \nolimits_{{t = 1}}^{M} \left( {{\text{log}}\frac{1}{{{\upvarepsilon}_{{\text{t}}} }}} \right){\text{f}}_{{\text{t}}} \left( {\mathbf{x}} \right)} {\mathop \sum \nolimits_{{t = 1}}^{M} \left( {{\text{log}}\frac{1}{{{\upvarepsilon}_{{\text{t}}} }}} \right)}}} \right. \kern-\nulldelimiterspace} {\mathop \sum \nolimits_{{t = 1}}^{M} \left( {{\text{log}}\frac{1}{{{\upvarepsilon}_{{t}} }}} \right)}};$$

where $\text{f}(\mathbf{x})$ is the final predicted value and ${\text{f}}_{t}(\mathbf{x})$ is the predicted value of the $t$-th weak learner; ${\upvarepsilon }_{t}$ is the error rate (${\upvarepsilon }_{t}={\overline{\text{L}} }_{t}/\left(1-{\overline{\text{L}} }_{t}\right)$); ${\overline{\text{L}} }_{t}$ is the average loss, and ${\overline{\text{L}} }_{t}=\sum\nolimits_{\text{i}=1}^{\textrm{m}}{\text{L}}_{t}(\text{i}){\textrm{D}}_{t}(\textrm{i})$, where ${\text{L}}_{t}(\text{i})$ is the error between the predicted value and the true value (i.e. corrected phenotype) of the i-th individual, and ${\text{D}}_{t}(\text{i})$ is the distribution of weights and ${\text{D}}_{t+1}\left(\text{i}\right)=\frac{{\text{D}}_{t}(\text{i}){\upbeta }_{t}^{(1-{\text{L}}_{t}(\text{i}))}}{{\text{Z}}_{t}}$, where ${\text{Z}}_{t}$ is the normalization factor such that the sum of ${\text{D}}_{t+1}\left(\text{i}\right)$ is 1. In this study, to reduce the hyperparameter tuning time and to reuse the experience of some of the used learners, KRR was used as the base learner for AdaBoost.R2 (abbreviated as AdaBoost.R2_KRR). When optimizing the hyperparameters of AdaBoost.R2, the optimal number of base learners was unstable and difficult to determine. Therefore, we used the default number of base learners (i.e., 50).

For the above three ML methods, genomic prediction was performed with the help of the sklearn package for Python (V0.22). In addition, considering that the optimal hyperparameters of ML methods might differ between populations, it is not reasonable to train the model for prediction by directly combining populations, especially for populations with different genetic backgrounds. Therefore, a population-specific hyperparameter optimization strategy based on cross-validation was adopted for all three ML methods. For the two-population scenario, first, the reference populations for genomic selection of each population were randomly divided into five groups, four of which were treated as the training set, and the remaining set was treated as the test set. Then, the training sets of the two populations were combined to construct a joint training set, the Pearson correlation between corrected phenotypes ${\mathbf{y}}_{\mathbf{c}}$ and predicted genetic effects of the test set under different hyperparameter combinations was calculated separately for each population by grid search, and the hyperparameter combination with the highest average Pearson correlation was used as the optimal hyperparameter when predicting this population. The same strategy was used for the multi-population scenario to divide the Austrian and A training but the other three Chinese populations were not divided but were directly added to the training sets. The optimal hyperparameters for multi-population genomic prediction in the two-population and multi-population scenarios are shown in Tables 2 and 3, respectively.

**Table 2:** Optimal hyperparameters for machine learning methods for two-population genomic prediction obtained through a grid search

Trait^a^	Method	Population	
		Austria	A
TNB	SVR	kernel = ‘rbf’, C = 4, gamma = 0.0001	kernel = ‘rbf’, C = 5, gamma = 0.0001
	KRR	kernel = ‘rbf’, $\lambda$ = 0.0001, gamma = 0.0001	kernel = ‘rbf’, $\lambda$ = 5, gamma = 0.0001
	Adaboost.R2	KRR_kernel = ‘rbf’, KRR_ $\lambda$= 0.001, KRR_gamma = 0.0001	KRR_kernel = ’rbf’, KRR_ $\lambda$ = 2.5, KRR_gamma = 0.0001
NBA	SVR	kernel = ‘rbf’, C = 4, gamma = 0.0001	kernel = ‘rbf’, C = 5, gamma = 0.0001
	KRR	kernel = ‘rbf’, $\lambda$ = 0.0001, gamma = 0.0001	kernel = ‘rbf’, $\lambda$ = 3, gamma = 0.0001
	Adaboost.R2	KRR_kernel = ’rbf’, KRR_ $\lambda$ = 0.01, KRR_gamma = 0.0001	kernel = ’rbf’, KRR_ $\lambda$ = 2, KRR_gamma = 0.0001

^a^*TNB* total number of piglets born, *NBA* number of piglets born alive, *SVR* support vector regression, *KRR* kernel ridge regression

**Table 3:** The optimal hyperparameters for machine learning methods for multi-population genomic prediction obtained through a grid search

Trait^a^	Method	Population	
		Austria	A
TNB	SVR	kernel = ‘rbf’, C = 3, gamma = 0.0001	kernel = ‘rbf’, C = 1, gamma = 0.0001
	KRR	kernel = ‘rbf’, $\lambda$ = 5, gamma = 0.0002	kernel = ‘rbf’, $\lambda$ = 2, gamma = 0.0001
	Adaboost.R2	KRR_kernel = ‘rbf’, KRR_ $\lambda$ = 0.1, KRR_gamma = 0.0001	KRR_kernel = ’rbf’, KRR_ $\lambda$ = 0.1, KRR_gamma = 0.0001
NBA	SVR	kernel = ‘rbf’, C = 3, gamma = 0.0001	kernel = ‘rbf’, C = 5, gamma = 0.0001
	KRR	kernel = ‘rbf’, $\lambda$=4, gamma = 0.0002	kernel = ‘rbf’, $\lambda$ = 2, gamma = 0.00005
	Adaboost.R2	KRR_kernel = ’rbf’, KRR_ $\lambda$ = 0.05, KRR_gamma = 0.0001	kernel = ’rbf’, KRR_ $\lambda$ =0.05, KRR_gamma = 0.0001

^a^*TNB* total number of piglets born, *NBA* number of piglets born alive, *SVR* support vector regression, *KRR* kernel ridge regression

### Cross-validation and genomic predictive ability

Because of the inconsistency in birth years between the Austria and A populations (as shown in Table 1) and the small size of both populations, we did not adopt the strategy of using young individuals as the validation set. Instead, 10 replicates of fivefold cross-validation (CV) were performed to estimate the predictive ability, mean square error (MSE), and unbiasedness. For the two-population scenario, all individuals from each population were randomly divided into five groups, with four groups serving as the reference population and the remaining group as the validation population; the reference populations of the two populations were then combined to create a joint reference population. For the multi-population scenario, the same partitioning strategy was used for the Austrian and A reference sets but the other three Chinese populations were not divided; instead, they were directly added to the reference population. Notably, for both scenarios, the reference and validation populations for genomic prediction were the same for all methods for each replicate of the fivefold CV.

For all methods, predictive ability was assessed as the Pearson correlation between corrected phenotypes ${\mathbf{y}}_{\mathbf{c}}$ and predicted values (i.e., breeding values in GBLUP and BayesHE, and genetic effects including additive and non-additive effects in ML methods) of the validation population, prediction bias was calculated as the regression of ${\mathbf{y}}_{\mathbf{c}}$ on predicted values of the validation population, and MSE was computed as follows:

$$\text{MSE}=\frac{1}{\text{n}}{{\sum }_{\text{i}=1}^{\text{n}}({\text{f}}_{\text{i}}^{\prime}-{\text{y}}_{\text{ci}}^{\prime})}^{2} ,$$

where $\text{n}$ represents the number of animals in each validation data set of the fivefold CV, and the vectors ${\mathbf{f}}^{\mathbf{^{\prime}}}$ and ${\mathbf{y}}_{\mathbf{c}}^{\mathbf{^{\prime}}}$ represent the centralized predicted values (i.e., predicted value minus the mean of the validation population) and centralized observed values (i.e., corrected phenotype minus the mean of the validation population), respectively. The overall predictive ability, MSE, and unbiasedness were the averages of 10 replicates of fivefold CV. In addition, for all evaluation metrics (predictive ability, MSE and unbiasedness), the standard error of the fivefold CV results for each replicate was calculated, and the final standard error reported was the average of 10 replicates. To compare the prediction accuracy of different methods, multiple t-tests (with P-values adjusted using the Bonferroni method) were conducted based on the outcomes of 10 replicates.

## Results

### Population structure and genetic parameters

Due to the lack of genetic exchange between the five pig populations, no pedigree connections between the different populations were observed. However, the PCA SNP genotypes of the five populations (Fig. 1b) showed that the genetic backgrounds of the Austrian and A pigs are relatively closer than those of the other three Chinese populations.

Table 4 illustrates the LD between adjacent SNPs across populations. The average ${\text{r}}_{\text{LD}}^{2}$ of adjacent SNPs on each chromosome in the Austrian, A, B, C, and D populations ranged from 0.33 to 0.42, 0.34 to 0.43, 0.34 to 0.44, 0.34 to 0.44, and 0.34 to 0.45, respectively, and the average ${\text{r}}_{\text{LD}}^{2}$ across all chromosomes in the Austria, A, B, C, and D populations were equal to 0.37, 0.35, 0.38, 0.39, and 0.40, respectively. The average correlation of ${\text{r}}_{\text{LD}}$ (i.e., LD consistency) between adjacent SNPs across all chromosomes was 0.41, 0.32, 0.35, and 0.34 for Austria with populations A, B, C, and D, respectively; for population A, the average correlation of ${\text{r}}_{\text{LD}}$ with populations B, C, and D was 0.29, 0.36, and 0.35, respectively. The correlations of ${\text{r}}_{\text{LD}}$ also suggested the genetic relationship between the Austria and A populations is closer than that between the Austria and other populations.

**Table 4:** Linkage disequilibrium between adjacent SNPs by chromosome and populations

Chr	Length (Mb)	Number of SNPs	Mean distance (kb)	Mean ${\text{r}}_{\text{LD}}^{2}$					Cor^a^						
				Austria^c^	A^c^	B^c^	C^c^	D^c^	Aus-A	Aus-B	Aus-C	Aus-D	A−B	A−C	A−D
1	261.26	5177	48.14	0.41	0.41	0.44	0.44	0.44	0.49	0.29	0.42	0.42	0.29	0.39	0.41
2	144.32	3133	44.59	0.37	0.39	0.39	0.39	0.39	0.30	0.31	0.37	0.36	0.26	0.29	0.33
3	126.4	2849	42.51	0.36	0.36	0.37	0.39	0.39	0.51	0.34	0.36	0.28	0.21	0.36	0.35
4	124.7	2851	38.9	0.41	0.41	0.40	0.44	0.45	0.41	0.29	0.34	0.34	0.29	0.32	0.31
5	99.43	2222	43	0.35	0.36	0.36	0.37	0.38	0.40	0.38	0.35	0.34	0.29	0.45	0.39
6	162.85	2839	53.6	0.40	0.40	0.42	0.41	0.42	0.45	0.36	0.40	0.44	0.36	0.41	0.42
7	115.97	2914	38.11	0.34	0.35	0.36	0.36	0.37	0.41	0.31	0.29	0.28	0.30	0.32	0.35
8	132.37	2871	43.73	0.34	0.36	0.36	0.37	0.38	0.46	0.32	0.33	0.33	0.35	0.33	0.29
9	132.86	3026	41.98	0.37	0.39	0.37	0.41	0.41	0.51	0.32	0.46	0.36	0.27	0.41	0.43
10	65.89	1832	34.64	0.33	0.34	0.36	0.34	0.34	0.40	0.31	0.37	0.38	0.31	0.38	0.34
11	75.35	1773	40.78	0.33	0.34	0.35	0.36	0.36	0.36	0.22	0.36	0.22	0.21	0.37	0.37
12	58.11	1719	33.2	0.39	0.40	0.38	0.41	0.41	0.45	0.25	0.32	0.28	0.25	0.27	0.26
13	198.36	3795	49.51	0.42	0.43	0.43	0.44	0.44	0.51	0.49	0.50	0.48	0.33	0.47	0.47
14	135.07	3359	38.37	0.41	0.41	0.41	0.44	0.44	0.37	0.30	0.26	0.31	0.30	0.33	0.29
15	133.57	2739	44.63	0.40	0.40	0.40	0.43	0.43	0.32	0.33	0.35	0.41	0.32	0.32	0.38
16	75.58	1776	39.79	0.34	0.35	0.34	0.36	0.37	0.29	0.27	0.31	0.30	0.19	0.35	0.25
17	59.77	1605	36.19	0.34	0.34	0.34	0.37	0.38	0.35	0.26	0.25	0.25	0.40	0.28	0.35
18	53.08	1254	39.22	0.34	0.36	0.37	0.36	0.37	0.36	0.28	0.34	0.37	0.25	0.40	0.38
Mean	–	–	41.72	0.37	0.35	0.38	0.39	0.40	0.41	0.32	0.35	0.34	0.29	0.36	0.35
Total	2154.94^b^	47.734^b^	–	–	–	–	–	–	–						

^a^Austria-A/B/C/D: the correlation of ${r}_{LD}$ of pairs of adjacent SNPs between the Austrian and A/B/C/D populations; A-B/C/D: the correlation of ${r}_{LD}$ of pairs of adjacent SNPs between the A and B/C/D populations

^b^Across the 18 porcine autosomes

^c^Yorkshire pig populations from Austria, A, B, C and D

Pedigree-based estimates of heritability for each population using the single-trait repeatability model during the derivation of corrected phenotypes are in Table 1. The heritability estimates for TNB and NBA ranged from 0.05 to 0.12 in the five populations.

### Genotype imputation accuracy

**Fig. 2:**
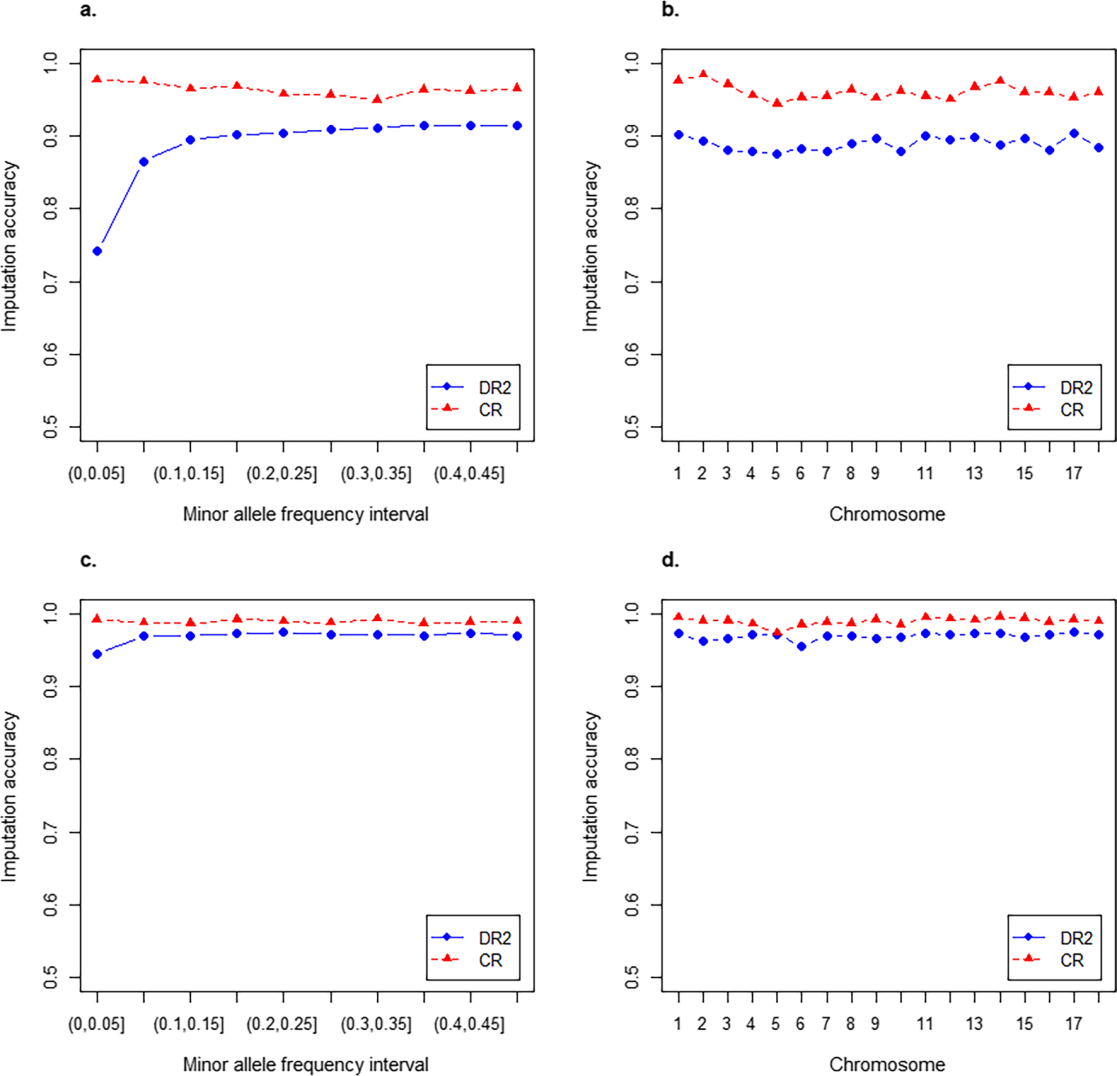
Average imputation accuracy for SNPs with different minor allele frequency (MAF) intervals and chromosomes. **a**, **b** Average imputation accuracy of Austria 50 K genotypes to GenoBaits Porcine SNP 50 K. **c**, **d** Average imputation accuracy of the PorcineSNP50 BeadChip genotypes to GenoBaits Porcine SNP 50 K. DR2: average estimated squared correlation between the imputed and the true allele dose; genotype concordance rate (CR): average ratio of the correctly imputed genotypes

The imputation accuracy at different MAF intervals and for each chromosome is illustrated in Fig. 2. DR^2^ was sensitive to MAF when the Austria 50 K genotypes were imputed to GBTS50K and was relatively low for markers with a MAF lower than 0.05 (Fig. 2a). No significant differences in DR^2^ and CR were observed between chromosomes (Fig. 2b, d). Overall, the average DR^2^ values of the Austria 50 K and PorcineSNP50 genotypes imputed to GBTS50K were 0.89 and 0.97, respectively, while the average CR of the Austria 50 K and PorcineSNP50 genotypes imputed to GBTS50K was 0.96 and 0.99, respectively. Accordingly, both imputations were sufficiently accurate to analyse the three SNP panels together.

### Genomic prediction in the two-population scenario

#### Comparison of ML methods with other methods

**Fig. 3:**
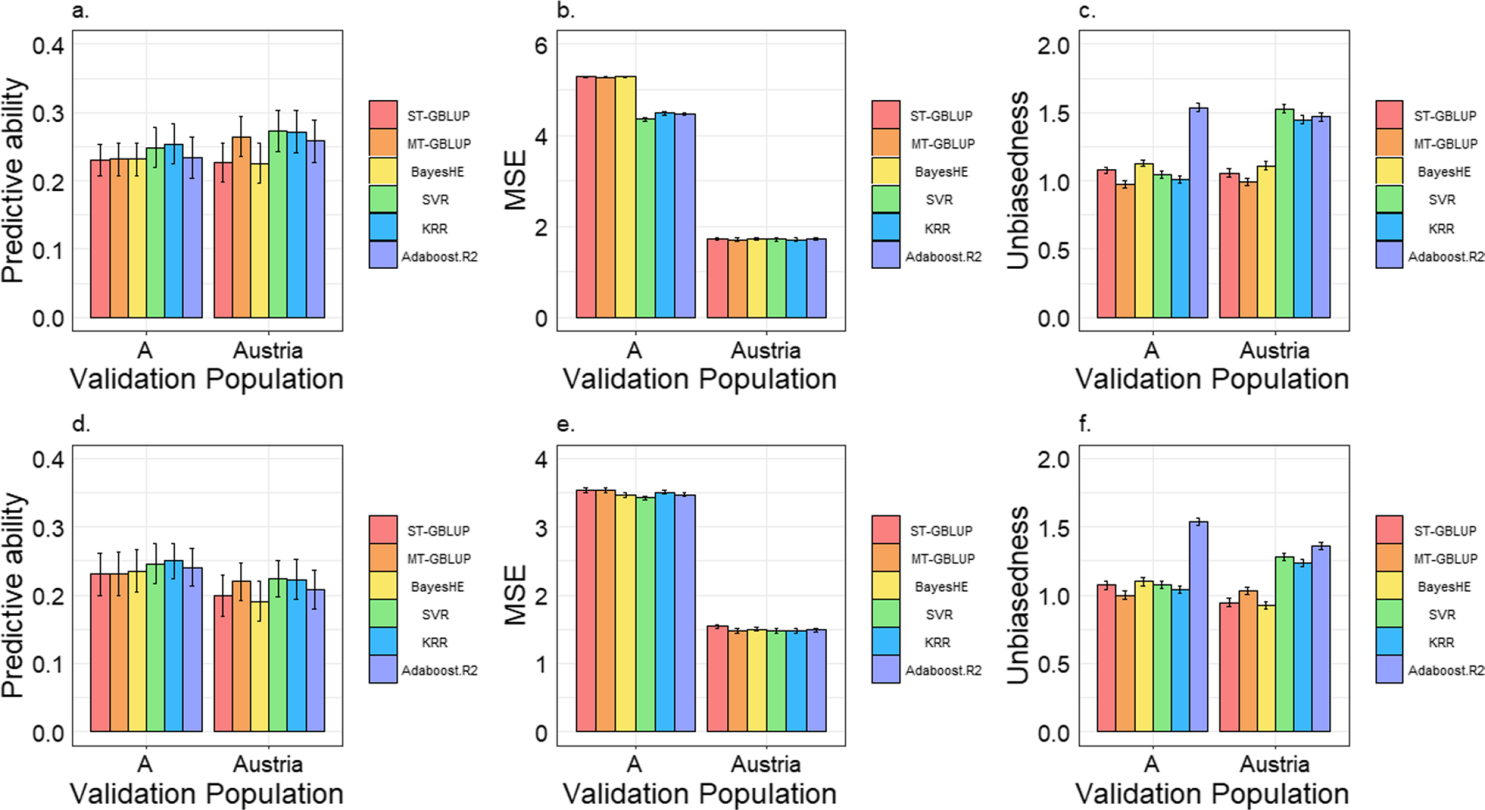
Predictive ability, mean squared error (MSE), and bias for genomic predictions for two populations with genetically linked backgrounds. **a**–**c** Predictive ability, MSE, and bias for total number of piglets born (TNB). **d**–**f** Predictive ability, MSE, and bias for number of piglets born alive (NBA). ST-GBLUP: single-trait genomic best linear unbiased prediction model; MT-GBLUP: multitrait GBLUP model. The error bar represents the standard error

Figure 3 presents the average predictive ability, MSE and unbiasedness values for the TNB and NBA traits in the two-population scenario (the raw values of Fig. 3 are in Additional file 1: Table S3). In all population and trait combinations, the highest predictive abilities were achieved using SVR and KRR. However, the predictive ability of AdaBoost.R2 was not significantly higher than that of MT-GBLUP for all traits and populations. When SVR, KRR, and Adaboost.R2 were used, improvements in average predictive ability were 4.5, 5.3, and −0.8% (averaged for two traits and two populations), respectively, compared to MT-GBLUP and 11.9, 12.6, and 6.1% (averages of both traits and both populations), respectively, compared to ST-GBLUP. The average improvement in predictive ability of the ML methods compared to ST-GBLUP, MT-GBLUP, and BayesHE were 10.2, 3.0, and 10.8%, respectively (averages of both traits and both populations). Although the predictive abilities of SVR and KRR were higher than those of MT-GBLUP in all population and trait combinations, this superiority was significant only for the A population and not for the Austrian population. Compared to MT-GBLUP, the improvement obtained with ML was greater for population A than for the Austrian population, while compared to ST-GBLUP, it was smaller for population A than for the Austrian population. Compared to MT-GBLUP, SVR, and KRR produced lower MSE in most scenarios, while Adaboost.R2 resulted in lower MSA than MT-GBLUP only for A population. In terms of unbiasedness, in the A population, AdaBoost.R2 generated the largest bias for both TNB and NBA traits, while the other methods yielded small biases (i.e., the regression coefficients were close to 1); in the Austria population, the biases of all three ML methods were much larger than those of ST-GBLUP, MT-GBLUP, and BayesHE.

#### Comparison of ST-GBLUP, MT-GBLUP and BayesHE

We found that the predictive ability was higher when MT-GBLUP was used compared to ST-GBLUP, with average improvements of 8.8 and 5.5% for TNB and NBA, respectively. Bias of predictions from MT-GBLUP were also closer to 1 (i.e., smaller bias) than for ST-GBLUP and MT-GBLUP yielded smaller MSE.

As shown in Fig. 3, the predictive ability of ST-GBLUP was similar to that of BayesHE for any population and none of these differences were statistically significant. However, MSE for TNB prediction were lower for ST-GBLUP than for BayesHE, but the opposite was observed for NBA (see Additional file 1: Table S3). For TNB, BayesHE resulted in a larger bias than ST-GBLUP (average regression values were 1.12 and 1.07, respectively) but for NBA, the biases were similar for these two methods.

In addition to the comparison of prediction performance, estimates of the genetic correlation estimated using MT-GBLUP between the Austrian and A populations were 0.62 and 0.40 for TNB and NBA, respectively, with standard errors of 0.125 and 0.152, respectively (see Additional file 1: Table S2).

### Genomic prediction in the multi-population scenario

#### Comparison of different methods

**Fig. 4:**
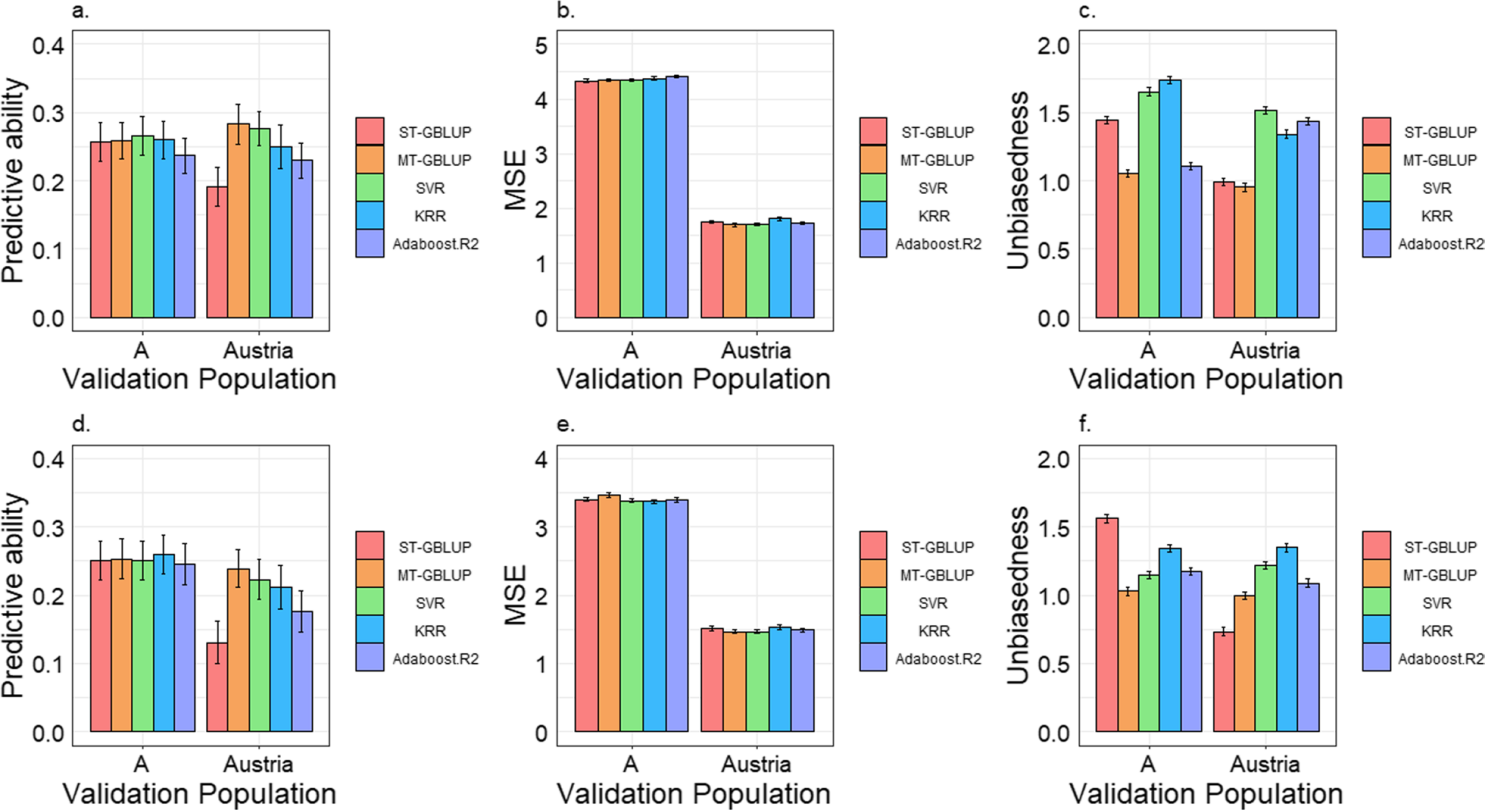
Predictive ability, mean squared error (MSE), and bias for genomic predictions for multiple populations with different genetic backgrounds. **a**–**c** Predictive ability, MSE, and bias for total number of piglets born (TNB). **d**–**f** Predictive ability, MSE, and bias for number of piglets born alive (NBA). ST-GBLUP: single-trait genomic best linear unbiased prediction model; MT-GBLUP: multitrait GBLUP model. The error bar represents the standard error

The genomic predictive abilities when the reference population size was expanded to multiple populations are shown in Fig. 4 (the raw values of Fig. 4 are also in Additional file 1: Table S3). Our results indicate that the ML methods did not demonstrate an overall advantage over MT-GBLUP in terms of predictive ability. Compared to MT-GBLUP, the predictive abilities of SVR and KRR improved by on average 1.0 and 1.6%, respectively, in A population, while they decreased by on average 4.4 and 11.5%, respectively, in the Austrian population (averages of both traits); Adaboost.R2 did not show any improvement in predictive ability compared to MT-GBLUP for either trait and either population. However, compared to ST-GBLUP, ML methods generally showed higher predictive abilities; 29.7, 24.4, and 11.1% higher for SVR, KRR, and AdaBoost.R2, respectively (averages of both traits and both populations). Moreover, for both traits, the ML methods yielded greater improvements over ST-GBLUP for the prediction of the Austrian population than for that of the A population. This might be due to the fact that when the reference population is enlarged from two populations to multiple populations, the predictive ability of ST-GBLUP for the Austrian population decreased, while it slightly increased for the A population. When predicting the Austrian population, average improvements of 57.6, 46.4 and 27.4% (averages of both traits) over ST-GBLUP were observed for SVR, KRR and AdaBoost.R2, respectively. However, when predicting the A population, the improvements were small, at 1.8 and 2.4% (averages of both traits) (averages of both traits) for SVR and KRR, respectively, while a decrease of 5.1% (averages of both traits) was observed for Adaboost.R2 compared to ST-GBLUP. It should also be noted that the predictive abilities for the A population were higher than those for the Austrian population for almost all traits and methods, as shown in Fig. 4.

In terms of MSE, MT-GBLUP produced the lowest MSE for most traits and populations; SVR and AdaBoost.R2 performed similar to or better than ST-GBLUP, while KRR produced higher MSE than ST-GBLUP for most traits and populations. Concerning bias, MT-GBLUP was unbiased, with values close to 1, for all population and trait combinations. In contrast, the other methods exhibited significant deviations from 1 for almost all scenarios. The ML methods resulted in larger biases than ST-GBLUP for all trait and population combinations, except for NBA in the A population, which resulted in regression coefficients closer to 1, although still relatively large (> 1.14).

#### Comparison of the two-population and multi-population scenarios

Figures 3 and 4 show the predictive abilities and MSE in the two-population and multi-population scenario, respectively, with the underlying values reported in Additional file 1: Table S3. When enlarging the reference population of genomic prediction from two to multiple populations, ST-GBLUP, KRR, and Adaboost.R2 all improved predictive ability in population A (with average improvements of, respectively, 10.2, 3.2, and 1.5% across the two traits), while their predictive ability decreased in the Austrian population (with an average decrease of, respectively, 25.0, 6.5, and 13.1% across the two traits). When enlarging the reference population, MT-GBLUP showed an improvement in predictive ability for all population and trait combinations, and this improvement was statistically significant for most population and trait combinations. Although improvements in predictive ability when moving from two-populations to multi-populations were generally also observed for SRV (average improvement of 2.5% across all populations and traits), statistical significance was only found for the TNB in population A (improvement of 6.83%). In addition, lower MSE were observed when using MT-GBLUP and SVR for multiple populations than for two populations for all trait and population combinations but similar or lower MSE were obtained when using ST-GBLUP, KRR, and AdaBoost.R2 for multiple populations than for two populations. Moreover, when moving from two populations to multiple populations, prediction bias generally increased for ST-GBLUP and the ML methods, but remained approximately the same for MT-GBLUP.

## Discussion

The predictive ability of multi-population genomic prediction is affected by a variety of factors, such as differences in the LD between markers and quantitative trait loci (QTL) across populations, QTL segregating in one population only and not the other, differences in MAF between populations, and differences in allele substitution effects due to the different backgrounds of the populations [39, 40]. In this study, we explored the effectiveness of joint genomic prediction in Chinese and Austrian pigs in a two-population scenario and a multi-population scenario.

As Robertson [41] suggested, 0.80 is the biologically important threshold for G × E interactions. The genetic correlation for the TNB and NBA traits between the Austria and A populations were 0.62 and 0.40, respectively (see Additional file 1: Table S2). Therefore, G × E interactions are likely to exist between the two populations. In the two-population scenario, multitrait models can be used to improve the predictive ability of genomic prediction due to their ability to capture the G × E interactions between populations, but higher predictive ability can be further gained using ML methods. In the multi-population scenario, apart from the Austria and A populations, populations C and D also had similar genetic backgrounds to each other (Fig. 1b) but the genetic correlations between them were lower than those between the Austria and A populations (see Additional file 1: Table S2), suggesting the possibility of G × E interactions between the C and D populations as well. Considering that large G × E interactions were not accounted for in single-trait models, ST-GBLUP also achieved an overall lower predictive ability compared to MT-GBLUP and ML methods, and MT-GBLUP resulted in a smaller bias than other methods.

In the two-population scenario, compared to ST-GBLUP, the predictive ability of MT-GBLUP improved by on average 8.8 and 5.5% for TNB and NBA, respectively. In addition, multitrait models can also be used to account for different phenotype scales between populations, particularly in the context of international joint evaluations, in which traits are not defined in the same way and country-specific scale effects may apply [42]. Nevertheless, the predictive abilities of SVR and KRR were higher than that of MT-GBLUP in almost all cases (Fig. 3). In the multi-population scenario, MT-GBLUP is also an ideal “benchmark” for comparison, but it failed to converge when the covariances between populations were estimated using a 5-trait model. Therefore, a series of bivariate analyses were performed and the estimated genetic parameters were combined into the final (co)variance matrix. This approach of using a series of bivariate analyses is commonly practised in international dairy and beef evaluations to deal with multiple populations [43]. Alternative approaches could be used to estimate (co)variance components, such as a Bayesian sampling approach (e.g., Gibbs sampling) instead of the REML method [44] that was used here. When using multitrait models to handle multiple populations, in spite of genetic differences populations, those with similar genetic backgrounds can be collectively modelled as a single population, which reduces the number of traits in the model, significantly reducing computational demands. The same approach is applicable in scenarios involving different breeds or G × E interactions, since farms from similar areas tend to exhibit fewer G × E interactions and can thus be regarded as a joint population. Therefore, multitrait models are still feasible when dealing with data from numerous populations.

When comparing MT-GBLUP with ML methods, in the two-population scenario, ML methods demonstrated superior predictive abilities with similar or smaller biases compared to MT-GBLUP in the A population, while they showed comparable predictive abilities to MT-GBLUP for the Austrian population but with increased biases; in the multi-population scenario, ML methods did not show higher predictive abilities than MT-GBLUP but greater prediction bias. Finally, the ML methods exhibited greater computational efficiency, particularly for the multi-population scenario, as shown in Additional file 1: Table S4. In addition, when the number of populations increased, only MT-GBLUP yielded an improvement in predictive ability in both populations, while the other methods yielded an improvement only in population A and not in the Austrian population. This could be because the added populations had significantly lower genetic correlations with the Austrian population compared to their correlations with population A (as shown in Additional file 1: Table S2); thus, they may have greater G × E interactions with the Austrian population. If G × E interactions are not adequately accounted for, adding such populations in the multi-population scenario might decrease the predictive ability. However, the only model that was proficient in handling this multi-population scenario is MT-GBLUP, as it can account for such G × E interactions. Consequently, only MT-GBLUP improved the predictive ability for the Austrian population in the multi-population scenario.

In this study, we did not find consistency between PCA and the genetic correlation of specific traits. In spite of the distinct genetic backgrounds of populations A, B, and C based on PCA (Fig. 1b), the estimates of genetic correlations for TNB and NBA between them were high (see Additional file 1: Table S2). The estimates of these genetic correlations may, however, be biased because the genetic markers may not accurately represent differences in allele frequencies at causal loci between populations [45]. Wientjes et al. [46] demonstrated that unbiased estimates of genetic correlation can be obtained from genomic relationships based on causal loci. However, when the non-causal SNPs that are used to estimate genetic correlation between populations do not have similar properties as the causal loci (e.g., similar pattern of allele frequencies), estimates of the genetic correlation can be biased [45]. Estimates of genetic correlations between populations can also be affected when the genetic effects captured by the genotyped markers have higher or lower genetic correlations than the portion that is not captured by markers [47]. Finally, genetic correlations differ across traits [48–50] and are also affected by potential differences in the environments that the populations are exposed to [51].

Among the three ML methods, SVR and KRR had higher predictive ability than ST-GBLUP. These findings were consistent with other studies, showing the superiority of SVR and KRR in terms of predictive ability for genomic prediction of phenotypes [16, 18, 52, 53]. ML methods have demonstrated an ability to use nonadditive effects and improve the predictive ability [16]. Moreover, the ML methods employed in this study adopted a population-specific hyperparameter optimization strategy, ensuring that the most suitable hyperparameters could be obtained for each population. In addition, the optimal hyperparameters were determined using a grid search based on the Pearson correlation coefficient between corrected phenotypes ${\mathbf{y}}_{\mathbf{c}}$ and predicted genetic effects, thereby ensuring that the identified optimal hyperparameters were closer to the global optimum. These factors contribute to the advantages of ML algorithms over ST(MT)-GBLUP and Bayesian methods in the two-population scenario. However, when adding unrelated reference populations, the data were not evenly distributed across PCA (Fig. 1b). The bandwidth parameter of the RBF kernel (i.e., the inverse of gamma) is affected by the distribution and local density of data points [54]. Therefore, using an RBF kernel with a fixed gamma value for all individuals may not adequately adapt to local features within the data, thereby struggling to establish connections between populations. In contrast, the MT-GBLUP method captures genetic connections between populations through the **G** matrix. Consequently, MT-GBLUP still achieved higher predictive abilities in that scenario. For ML methods, developing an RBF kernel that dynamically adjusts the bandwidth for each data point based on local density (i.e., reducing bandwidth within clusters to capture more subtle local features, while increasing bandwidth in sparser areas between clusters to establish connections across different clusters) could enable the model to better adapt to local features and distributions of data, which may be beneficial for multi-population datasets.

ML methods also have limitations. (1) Traditional ML methods often fail to decompose variance components, making the calculation of heritabilities and genetic correlations a challenge. (2) ML methods often exhibit sensitivity to minor perturbations (a change or disturbance to the original data) and noise in the data, which could lead to instability in the model output (as in the case of Adaboost.R2 in our study). (3) Hyperparameter optimization is required during the model training process. The time required for hyperparameter optimization with grid search is influenced by experience (i.e. the selection of the hyperparameter ranges and values in grid search) and novices may need some time to explore the optimal hyperparameters. However, some automated hyperparameter search strategies, such as random search, Bayesian optimization, and gradient-based hyperparameter optimization, can significantly improve optimization efficiency without being affected by experience. It should be noted that in practical breeding, hyperparameter optimization is not an issue because automatic hyperparameter optimization can be integrated into the analysis workflow. In addition, as long as there are no significant changes in the reference population, there is no need to re-optimize hyperparameters and previously trained models can be directly employed for prediction. And (4), traditional ML methods often struggle to facilitate practical selection to improve breeding values; because ML methods can capture nonlinear relationships in genomic data, the genetic effects that they predict also include non-additive effects (e.g., dominance and epistasis). Therefore, traditional ML methods are difficult to apply directly in practical applications for selection to improve breeding values, and more research is required to explore the dissection of additive effects.

Because the primary aim of this study was to compare the performance of phenotype prediction methods and because of limitations in sample size and birth year, a random CV strategy was adopted to achieve more stable results. However, in practical breeding, selection often requires using older animals in the training set to predict younger individuals. The predictive ability and bias obtained from CV may not represent those of practical predictive ability and bias because the relationship between the training and validation sets is different.

As an ensemble learning method, AdaBoost.R2 did not show advantages compared to SVR and KRR, for which there are three possible explanations. (1) The number of AdaBoost.R2 iterations (i.e., the number of base learners) has a strong impact on the predictive ability of the model [55]. However, the small size of each population and the differences between populations in the training set increased the differences between replicate training sets, thereby rendering the determination of the optimal number of base learners unstable and challenging (results not shown). As a result, we did not identify the optimal number of base learners but, instead, we used the default number of base learners, which may have somewhat compromised the performance of AdaBoost.R2. (2) Since AdaBoost.R2 mainly focuses on reducing bias through a stepwise boosting approach, it often overlooks variance control, leading to an increase in model overfitting. To increase the diversity of base learners and reduce the risk of overfitting, a strong ensemble must be constructed based on learners with fairly weak generalization performance (such as the classification and regression tree (CART) decision tree or the multi-layer perceptron (MLP) neural network) [55]. However, our choice to use the more robust KRR as the base learner for AdaBoost was primarily motivated by our desire to use fewer base learners and to capitalize on the tuning experience (i.e. the approximate range of hyperparameters) gained from the learners employed in this study (e.g., SVR and KRR), thereby significantly reducing hyperparameter optimization time. This may have resulted in a decrease in the predictive ability of AdaBoost.R2. And (3) The performance of AdaBoost.R2 is sensitive to abnormal samples, which refer to data points that significantly deviate from the norm or general distribution of the dataset, often due to measurement error or anomalies [55]. For multi-population genomic prediction, the individuals in the validation population and some of the individuals in the reference population come from different populations, and abnormal samples from different populations may be assigned greater weights in iterations, while samples from the same population may be assigned smaller weights, thus affecting the final predictive ability, which may also be the reason for the large bias observed for Adaboost.R2.

ML methods provide new options for multi-population genomic prediction. However, as the sample size increases, the computing time of traditional ML methods that model each population separately increases. In contrast, in transfer learning, previously trained models can be reused, and the knowledge learned in the source domain (e.g. large population) can be used to help learning tasks in the target domain (e.g. small population) according to the similarity of data, tasks (e.g. genomic prediction of phenotypes), and models; therefore, the model trained on big data can be transferred to small datasets successfully [56, 57].

## Conclusions

In this study, we used ST-GBLUP, MT-GBLUP, BayesHE, and three ML methods for multi-population genomic prediction of phenotypes reproductive traits in Chinese and Austrian pigs. Our results demonstrated that the MT-GBLUP method showed advantages over ST-GBLUP in both two-population and multi-population scenarios. When enlarging the reference population from two populations to multiple populations, ST-GBLUP and ML methods produced overall larger bias, while MT-GBLUP generally achieved similar bias. Compared to MT-GBLUP, ML methods possess potential to improve the genomic prediction ability of both populations in the two-population scenario, while in the multi-population scenario, the advantages of ML methods were not demonstrated.

### Supplementary Information

Supplementary material 1: Table S1. Number of generations, full-sib and half-sib families for each population when constructing the A matrix. Table S2. Estimates of genetic variance (block-diagonals), covariance (upper triangular blocks; italic) and genetic correlation (lower triangular blocks; in bold; standard errors in parentheses) of multi-trait genomic best linear unbiased prediction (MT-GBLUP). Table S3. Predictive ability, mean squared error (MSE), and unbiasedness of different methods in the two and multi-population scenarios. Table S4. Average computation time to complete each fold of fivefold cross-validation (CV) for all genomic prediction methods.

## Data Availability

The datasets used or analysed during the present study are available from the corresponding author on reasonable request.
