# Supplementary material for: Predictive ability of multi-population genomic prediction methods of phenotypes for reproduction traits in Chinese and Austrian pigs
Source: Genet Sel Evol. 2024 Jun 26;56:49. doi: 10.1186/s12711-024-00915-5 (PMC11201905; doi:10.1186/s12711-024-00915-5)
Supplement: Supplementary file 1 — Supplementary material 1: Table S1. Number of generations, full-sib and half-sib families for each population when constructing the A matrix. Table S2. Estimates of genetic variance (block-diagonals), covariance (upper triangular blocks; italic) and genetic correlation (lower triangular blocks; in bold; standard errors in parentheses) of multi-trait genomic best linear unbiased prediction (MT-GBLUP). Table S3. Predictive ability, mean squared error (MSE), and unbiasedness of different methods in the two and multi-population scenarios. Table S4. Average computation time to complete each fold of fivefold cross-validation (CV) for all genomic prediction methods. [file 12711_2024_915_MOESM1_ESM.docx]

**Table S1** Number of generations, full-sib and half-sib families for each population when constructing the **A** matrix

|  | Populations | | | | |
| --- | --- | --- | --- | --- | --- |
|  | Austria | A | B | C | D |
| Number of generations | 2 | 5 | 4 | 4 | 5 |
| Number of full-sibs | 93 | 323 | 699 | 493 | 637 |
| Number of half-sibs | 146 | 288 | 374 | 355 | 290 |

**Table S2** Estimates of genetic variance (block-diagonals), covariance (upper triangular blocks; italic) and genetic correlation (lower triangular blocks; in bold; standard errors in parentheses) of multi-trait genomic best linear unbiased prediction (MT-GBLUP).

| Trait^1^ | Population | Populations | | | | |
| --- | --- | --- | --- | --- | --- | --- |
|  |  | Austria | A | B | C | D |
| TNB | Austria | 0.772 | *0.530* | *0.302* | *0.271* | *-0.206* |
|  | A | **0.624**  (0.125) | 0.935 | *0.493* | *0.839* | *0.136* |
|  | B | **0.610**  (0.287) | **0.906**  (0.403) | 0.317 | *0.460* | *0.231* |
|  | C | **0.326**  (0.105) | **0.917**  (0.137) | **0.864**  (0.424) | 0.895 | *0.234* |
|  | D | **-0.408**  (0.414) | **0.245**  (0.463) | **0.715**  (0.491) | **0.432**  (0.284) | 0.328 |
| NBA | Austria | 0.511 | *0.254* | *0.265* | *0.161* | *-0.294* |
|  | A | **0.403**  (0.152) | 0.774 | *0.456* | *0.606* | *0.289* |
|  | B | **0.638**  (0.321) | **0.891**  (0.375) | 0.338 | *0.386* | *0.133* |
|  | C | **0.300**  (0.176) | **0.917**  (0.226) | **0.884**  (0.403) | 0.565 | *0.142* |
|  | D | **-0.512**  (0.322) | **0.408**  (0.387) | **0.285**  (0.487) | **0.235**  (0.232) | 0.647 |

^1^ TNB: total number of piglets born; NBA: number of piglets born alive;

**Table S3** Predictive ability, mean squared error (MSE), and unbiasedness of different methods in the two and multi-population scenarios

| Trait^1^ | Validation population | Measurement^2^ | ST-GBLUP^3^ | | MT-GBLUP^4^ | | BayesHE | SVR | | KRR | | Adaboost.R2 | |
| --- | --- | --- | --- | --- | --- | --- | --- | --- | --- | --- | --- | --- | --- |
|  |  |  | Two^5^ | Multi^6^ | Two^5^ | Multi^6^ | Two^5^ | Two^5^ | Multi^6^ | Two^5^ | Multi^6^ | Two^5^ | Multi^6^ |
| TNB | Austria | Predictive ability | 0.227±0.029^b^ | 0.191±0.028^a^ | 0.265±0.029^cd^ | 0.283±0.029^d^ | 0.226±0.030^b^ | 0.273±0.031^cd^ | 0.277±0.025^cd^ | 0.272±0.031^cd^ | 0.250±0.032^bc^ | 0.258±0.031^bc^ | 0.230±0.026^b^ |
|  |  | MSE | 1.730±0.129 | 1.753±0.118 | 1.711±0.122 | 1.696±0.122 | 1.732±0.129 | 1.715±0.136 | 1.697±0.128 | 1.712±0.125 | 1.811±0.121 | 1.728±0.123 | 1.729±0.131 |
|  |  | Unbiasedness | 1.059±0.167 | 0.991±0.164 | 0.994±0.189 | 0.953±0.123 | 1.111±0.178 | 1.529±0.201 | 1.518±0.175 | 1.446±0.184 | 1.341±0.173 | 1.471±0.201 | 1.440±0.188 |
|  | A | Predictive ability | 0.230±0.023^a^ | 0.257±0.029^bc^ | 0.232±0.024^a^ | 0.259±0.026^bc^ | 0.232±0.024^a^ | 0.249±0.029^b^ | 0.266±0.028^c^ | 0.254±0.029^bc^ | 0.260±0.027^bc^ | 0.234±0.030^a^ | 0.237±0.026^a^ |
|  |  | MSE | 5.282±0.269 | 4.334±0.248 | 5.279±0.270 | 4.340±0.238 | 5.283±0.270 | 4.350±0.228 | 4.347±0.242 | 4.482±0.230 | 4.377±0.246 | 4.465±0.221 | 4.409±0.244 |
|  |  | Unbiasedness | 1.078±0.138 | 1.446±0.191 | 0.973±0.133 | 1.052±0.120 | 1.129±0.155 | 1.045±0.168 | 1.653±0.221 | 1.011±0.172 | 1.736±0.213 | 1.538±0.207 | 1.110±0.188 |
| NBA | Austria | Predictive ability | 0.199±0.03^c^ | 0.131±0.031^a^ | 0.220±0.027^d^ | 0.239±0.028^e^ | 0.191±0.029^c^ | 0.224±0.027^d^ | 0.223±0.029^d^ | 0.223±0.029^d^ | 0.212±0.032^cd^ | 0.208±0.029^cd^ | 0.176±0.030^b^ |
|  |  | MSE | 1.546±0.086 | 1.514±0.091 | 1.481±0.086 | 1.463±0.09 | 1.501±0.089 | 1.479±0.090 | 1.465±0.088 | 1.477±0.089 | 1.528±0.090 | 1.489±0.090 | 1.490±0.089 |
|  |  | Unbiasedness | 0.945±0.154 | 0.734±0.179 | 1.030±0.196 | 0.997±0.127 | 0.927±0.154 | 1.280±0.177 | 1.225±0.172 | 1.235±0.173 | 1.350±0.180 | 1.361±0.192 | 1.087±0.196 |
|  | A | Predictive ability | 0.231±0.031^a^ | 0.251±0.029^b^ | 0.232±0.032^a^ | 0.253±0.029^b^ | 0.235±0.031^a^ | 0.246±0.029^ab^ | 0.251±0.029^b^ | 0.250±0.026^b^ | 0.260±0.029^c^ | 0.241±0.027^a^ | 0.245±0.030^ab^ |
|  |  | MSE | 3.539±0.18 | 3.406±0.188 | 3.533±0.179 | 3.468±0.181 | 3.467±0.189 | 3.426±0.195 | 3.384±0.192 | 3.511±0.185 | 3.374±0.181 | 3.473±0.182 | 3.396±0.185 |
|  |  | Unbiasedness | 1.073±0.166 | 1.562±0.205 | 0.998±0.171 | 1.028±0.122 | 1.100±0.175 | 1.078±0.138 | 1.149±0.140 | 1.038±0.141 | 1.342±0.151 | 1.535±0.179 | 1.174±0.157 |

^1^ TNB: total number of piglets born; NBA: number of piglets born alive;

^2^ Predictive ability: the correlation between predicted values and corrected phenotypic values in the validation population;

^3^ ST-GBLUP: single-trait GBLUP model;

^4^ MT-GBLUP: multi-trait GBLUP model;

^5^ Two: genomic prediction using a reference population composed of two populations;

^6^ Multi: genomic prediction using a reference population composed of five populations;

Different letters a, b, c, and d within the same row indicate significant difference as determined by multiple t-tests (P<0.05). (That is, the letters serve as symbols indicating the significance of differences between methods: if the difference between methods is significant (i.e., P<0.05), they will not share the same letter; if the difference is not significant (i.e., P>0.05), they will contain the same letter.)

**Table S4** Average computation time to complete each fold of 5-fold cross-validation (CV) for all genomic prediction methods.

| Method^a^ | Population^b^ | Trait^c^ | |
| --- | --- | --- | --- |
|  |  | TNB | NBA |
| ST-GBLUP | Two | 2min03s | 2min32s |
|  | Multi | 35min15s | 38min39s |
| MT-GBLUP | Two | 4min58s | 5min57s |
|  | Multi | 3h21min16s | 3h16min20s |
| BayesHE | Two | 1h54min38s | 2h7min45s |
| SVR | Two | 3min49s | 4min08s |
|  | Multi | 36min32s | 25min16s |
| KRR | Two | 1min08s | 1min25s |
|  | Multi | 3min26s | 3min03s |
| Adaboost.R2 | Two | 3min15s | 4min01s |
|  | Multi | 36min38s | 29min58s |

^a^ ST-GBLUP: single-trait GBLUP model; MT-GBLUP: multi-trait GBLUP model;

^b^ Two: genomic prediction using a reference population composed of two populations; Multi: genomic prediction using a reference population composed of five populations;

^c^ TNB: total number of piglets born; NBA: number of piglets born alive.

The running time of the methods was measured on an HP server (CentOS Linux 7.9.2009, 2.5 GHz Intel Xeon processor and 515 GB total memory).
